# Online Gerontology Courses: Issues of Equity

**DOI:** 10.1093/geroni/igab046.1352

**Published:** 2021-12-17

**Authors:** Anita Sharma

**Affiliations:** University of Louisiana at Monroe, Monroe, Louisiana, United States

## Abstract

The COVID-19 Pandemic changed higher education in several significant ways. The most significant impact was on methods of course delivery. In March 2020, all educational institutions changed their methods of instruction to fully online instruction. It happened almost overnight and left the students as well as the instructors unprepared for the unanticipated metamorphosis . The sudden and unanticipated change in the method of instruction and delivery of course contents also highlighted issues of equity. There appeared to be high levels of inequity in the use of technology across school and college campuses. A survey of students conducted by the author at the University of Louisiana at Monroe revealed different types of inequity such as, lack of finances to buy equipment, lack of training in the use of technology, and lack of personal space to study from home. A significant percentage of student population at ULM consists of first-generation college students. These students were impacted the most by the new methods of course delivery. Additionally, the author looked up similar surveys at other educational institutions and conducted a meta-analysis of published studies. This paper presents these findings.

